# Supplementary figures and images for: A sense of place: transcriptomics identifies environmental signatures in Cabernet Sauvignon berry skins in the late stages of ripening
Source: BMC Plant Biol. 2020 Jan 28;20:41. doi: 10.1186/s12870-020-2251-7 (PMC6986057; doi:10.1186/s12870-020-2251-7)

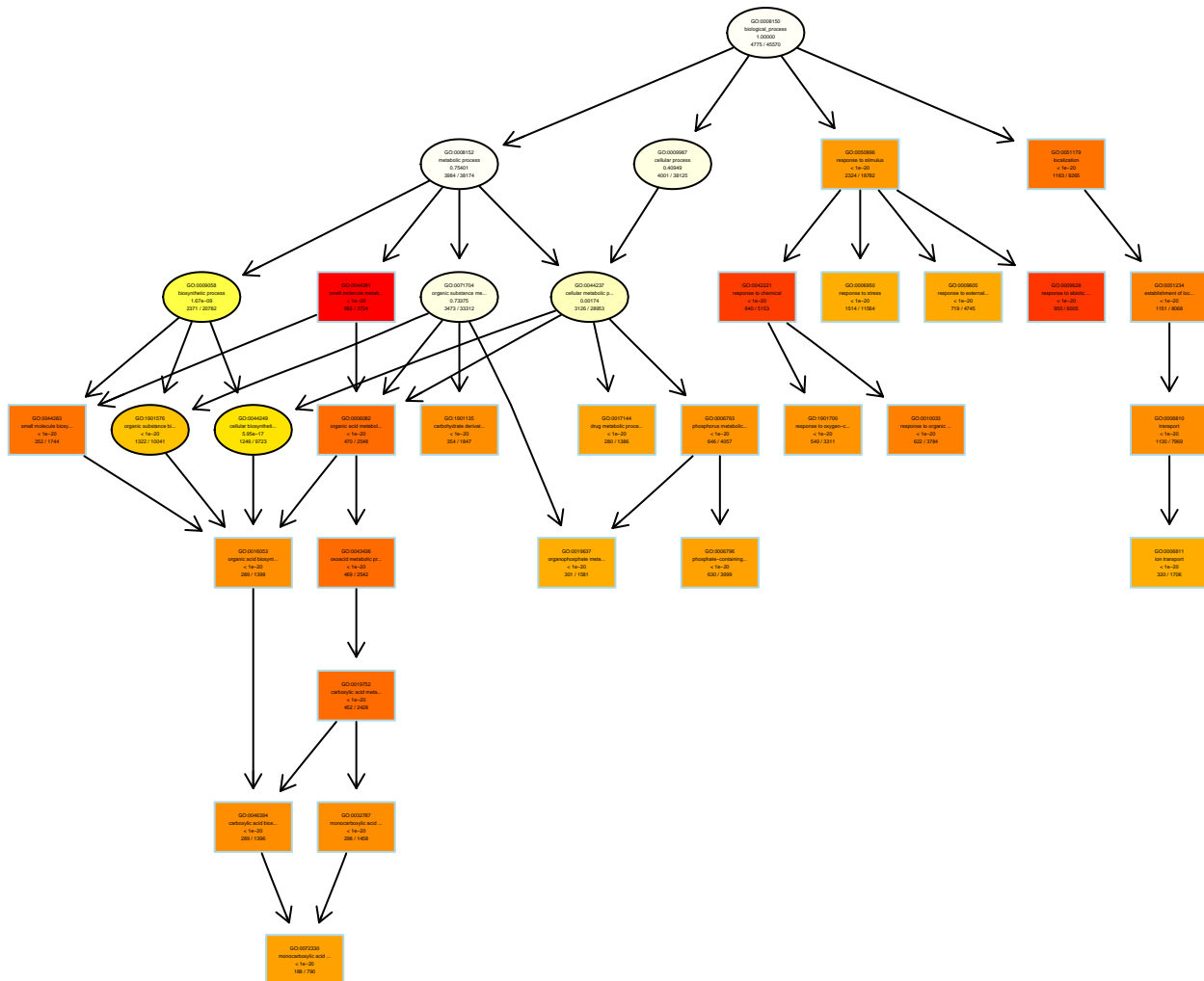

Supplement: Supplementary file 4 — Additional file 4. Image of the top 25 connected GO categories in the topGO network of the DEGs in Additional file 3. [file 12870_2020_2251_MOESM4_ESM.pdf]

# Module–trait relationships

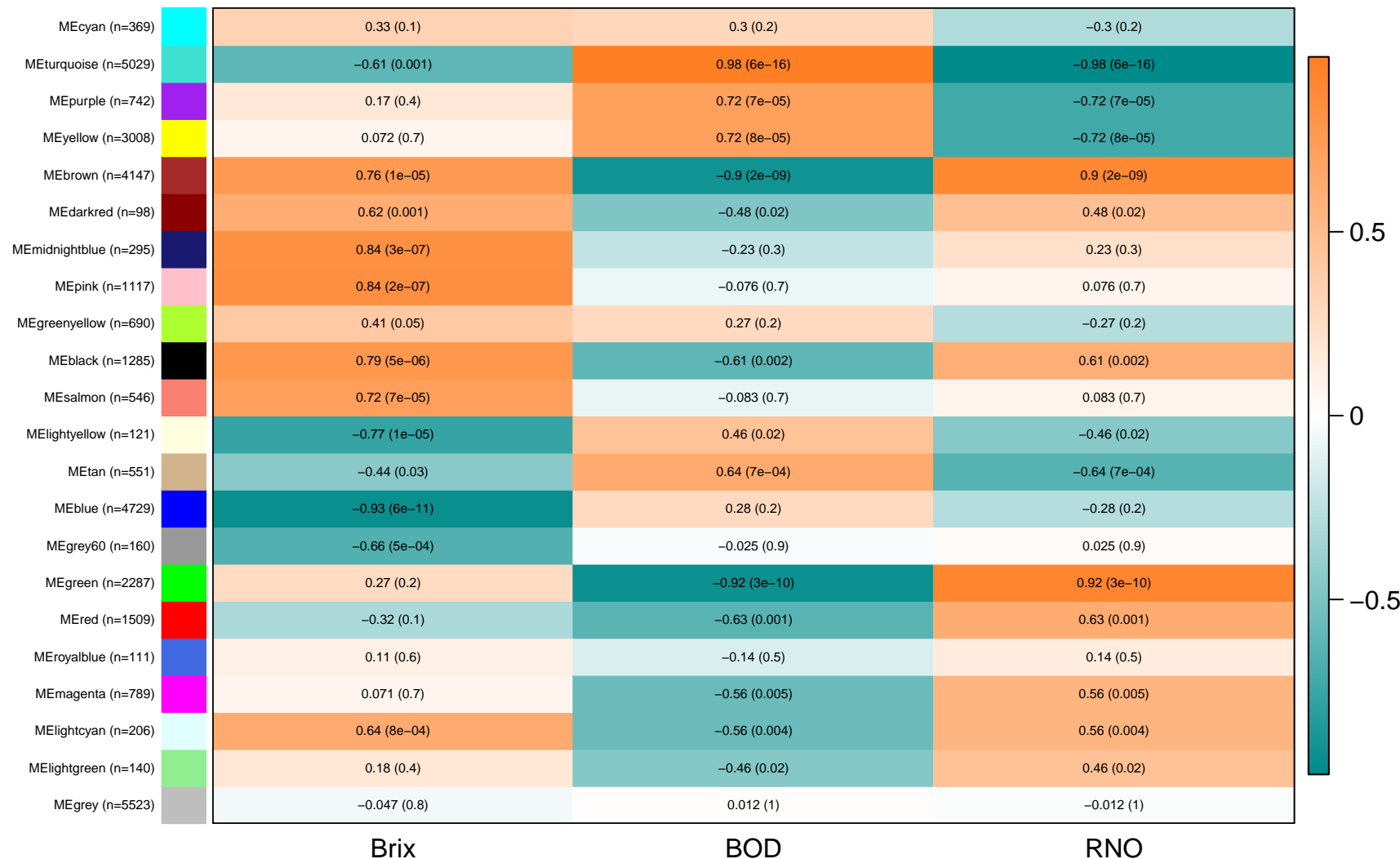

Supplement: Supplementary file 7 — Additional file 7 Heatmap correlation of berry traits (°Brix level, BOD, RNO) of each of 19 gene modules. Gene modules were identified by a color name (MMcolornumber) as assigned by the WGCNA R package. Values in each heatmap block are the correlation (left value) and p-value (in parentheses) of the module with the berry trait. [file 12870_2020_2251_MOESM7_ESM.pdf]

**STS10 (g455640)**

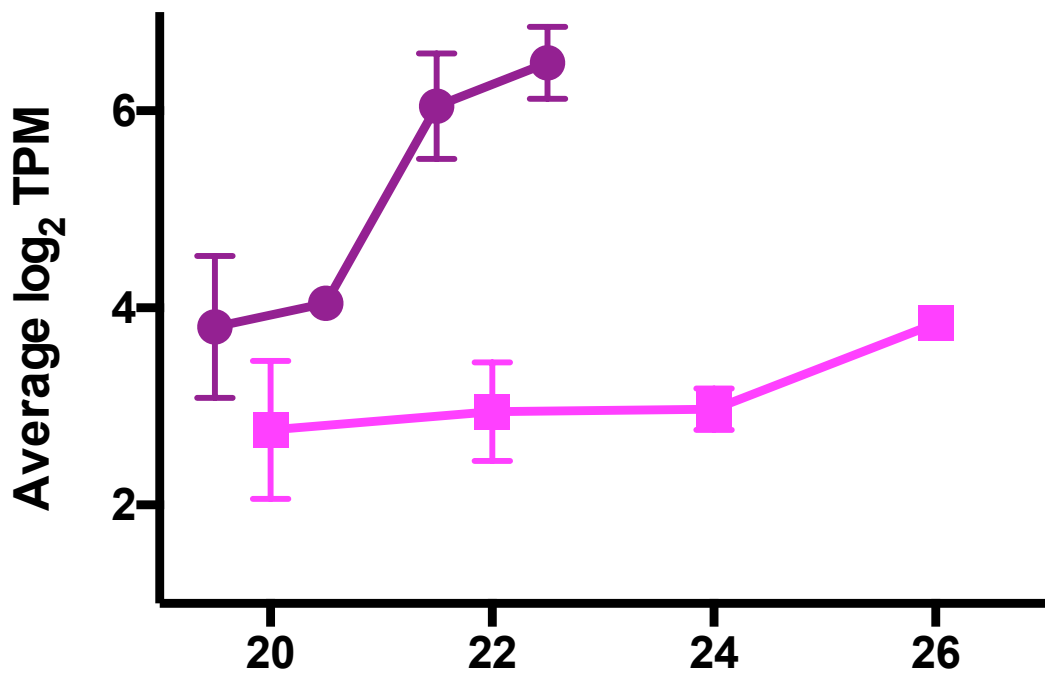

**STS47 (g199160)**

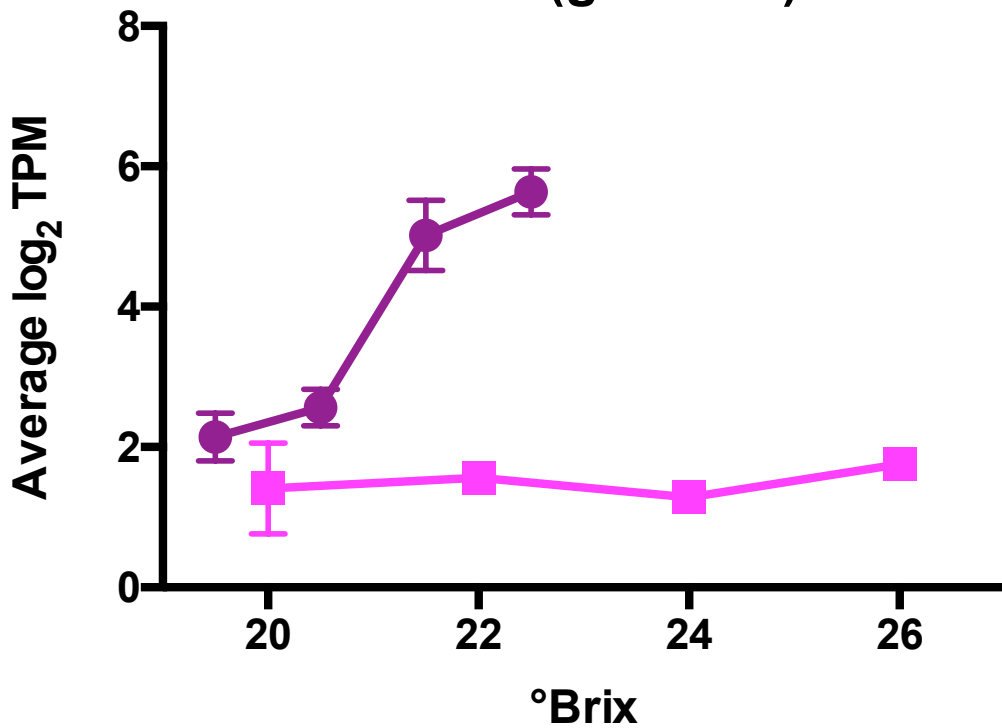

Supplement: Supplementary file 11 — Additional file 11. Representative examples of transcript profiles of some stilbene synthase (STS) genes that were differentially expressed. [file 12870_2020_2251_MOESM11_ESM.pdf]

Most core clock genes are very different between Reno and Bordeaux grown grapes

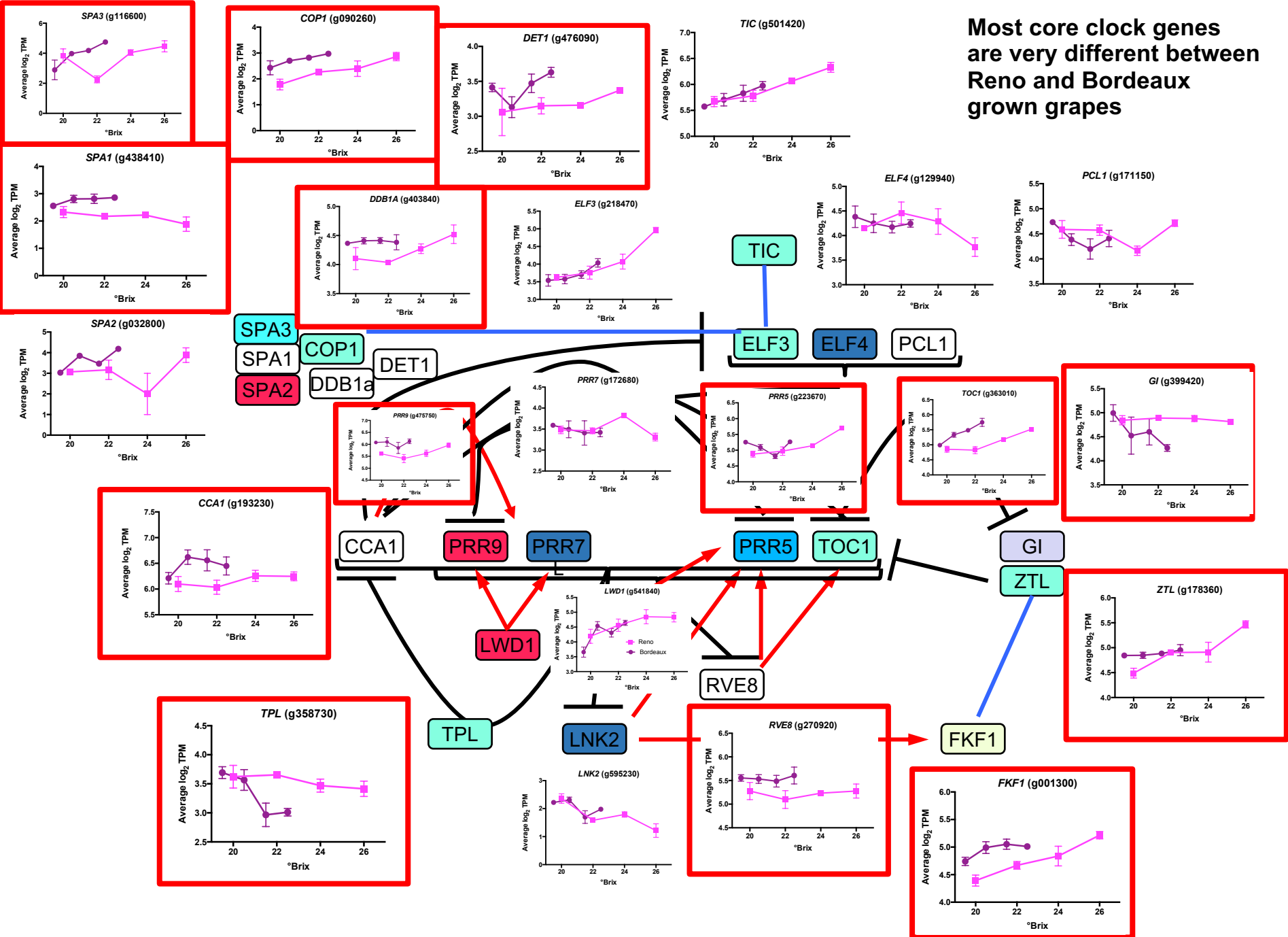

Supplement: Supplementary file 12 — Additional file 12. Transcript abundance of circadian clock genes from BOD and RNO berry skins. The data are placed on a circadian clock model derived from [4]. Lines in the model represent known interactions between genes; red arrows are positive interactions, black lines are negative interactions, and blue lines indicate direct physical interactions but the direction, positive or negative, is unknown. No lines indicate that there are no known interactions at this time. Transcript profiles outlined in red highlight significantly higher transcript abundance for the BOD berries. [file 12870_2020_2251_MOESM12_ESM.pdf]

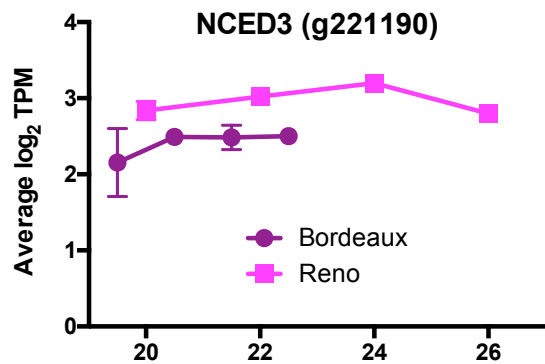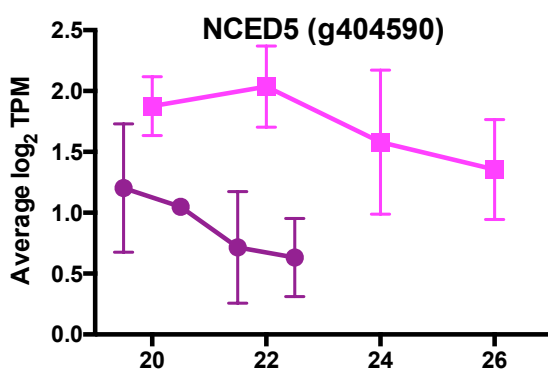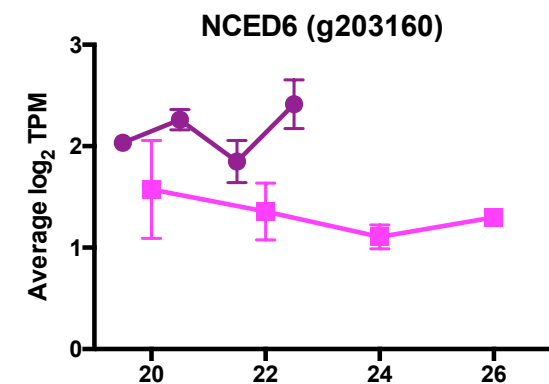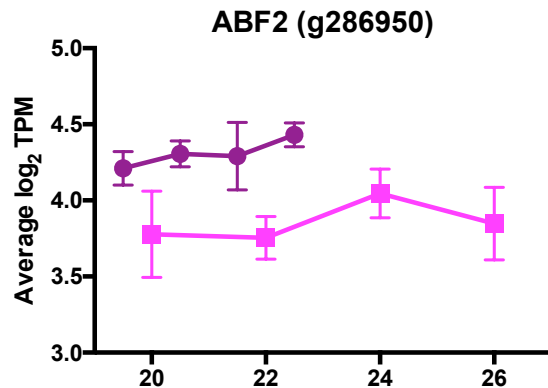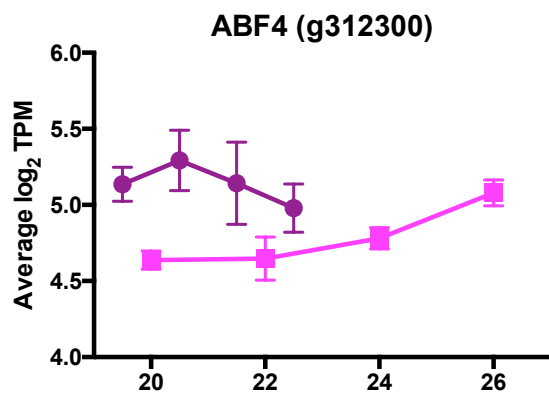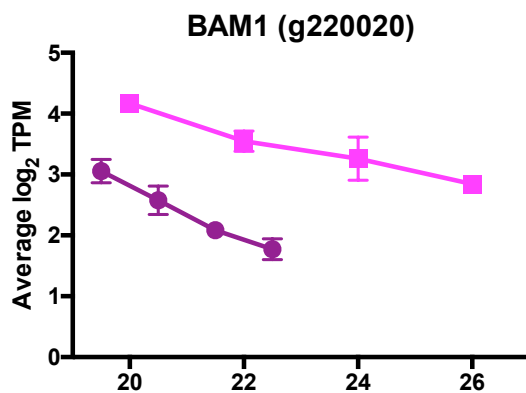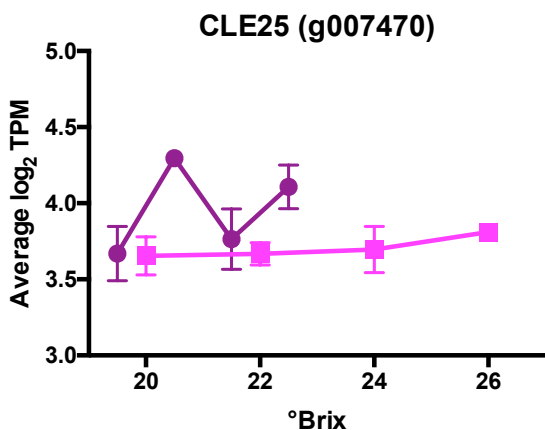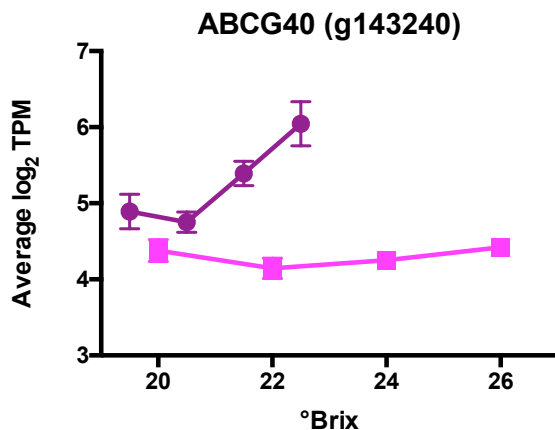

Supplement: Supplementary file 14 — Additional file 14. Transcript profiles of ABA biosynthesis and signaling genes that are differentially expressed between BOD and RNO berry skins. [file 12870_2020_2251_MOESM14_ESM.pdf]
