# Supplementary material for: A sense of place: transcriptomics identifies environmental signatures in Cabernet Sauvignon berry skins in the late stages of ripening
Source: BMC Plant Biol. 2020 Jan 28;20:41. doi: 10.1186/s12870-020-2251-7 (PMC6986057; doi:10.1186/s12870-020-2251-7)
Supplement: Supplementary file 13 — Additional file 13. A model of the peripheral genes including light sensing genes that interact with core circadian clock genes in BOD and RNO berry skins. Lines represent gene interactions as described in Additional file 12. Red and blue lightning bolts represent the reception of their respective light wavelengths for each gene symbol. Transcript profiles outlined in red highlight significantly higher transcript abundance for the BOD berries. [file 12870_2020_2251_MOESM13_ESM.pdf]

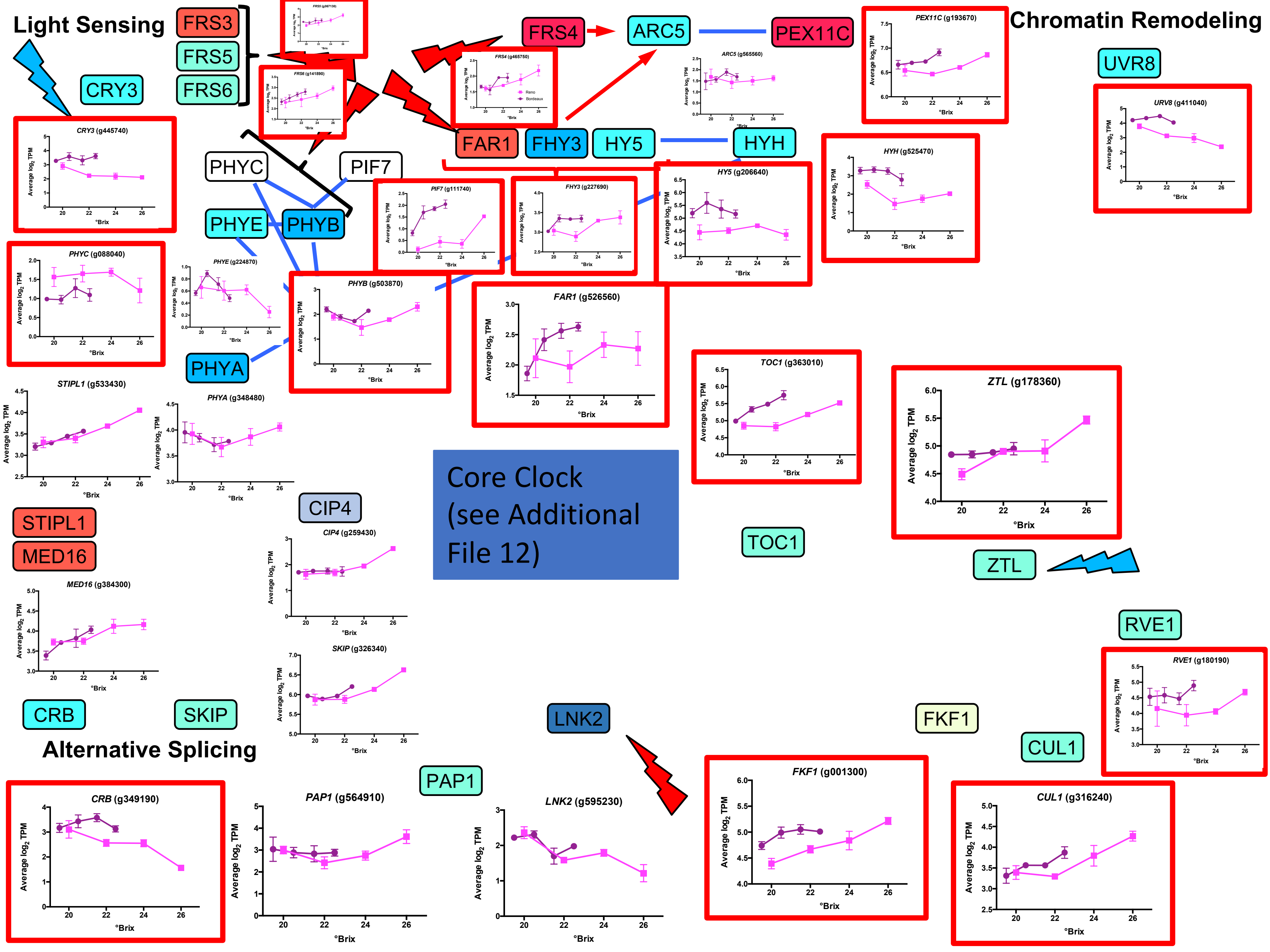

Light Sensing
FRS3
FRS5
FRS6
CRY3
FRS4
ARC5
PEX11C
Chromatin Remodeling
UVR8
FAR1
FHY3
HY5
HYH
PHYC
PIF7
PHYE
PHYB
PHYA
STIPL1
MED16
CIP4
Core Clock (see Additional File 12)
TOC1
ZTL
RVE1
FKF1
CUL1
LNK2
CRB
SKIP
PAP1
Alternative Splicing
CRY3 (g445740)
PHYC (g088040)
PHYE (g224870)
PHYB (g503870)
PIF7 (g111740)
FHY3 (g227690)
HY5 (g206640)
HYH (g525470)
URV8 (g411040)
PEX11C (g193670)
ARC5 (g565560)
FRS4 (g465750)
FRS6 (g141890)
FAR1 (g526560)
TOC1 (g363010)
ZTL (g178360)
STIPL1 (g533430)
PHYA (g348480)
CIP4 (g259430)
SKIP (g326340)
MED16 (g384300)
RVE1 (g180190)
FKF1 (g001300)
CUL1 (g316240)
CRB (g349190)
PAP1 (g564910)
LNK2 (g595230)
Reno
Bordeaux
Average log2 TPM
°Brix
